# Pepxml: ESM2-based extreme multilabel classification of pathogen-targeted antimicrobial peptides

**DOI:** 10.1093/bib/bbaf548

**Published:** 2025-10-17

**Authors:** Yannan Bin, Daijun Zhang, Zhiyang Hu, Chungui Xu, Yansen Su

**Affiliations:** The Key Laboratory of Intelligent Computing and Signal Processing of Ministry of Education, School of Life Sciences and Medical Engineering, Anhui University, Jiulong Road 111#, Hefei, Anhui 230601, China; The Key Laboratory of Intelligent Computing and Signal Processing of Ministry of Education, School of Life Sciences and Medical Engineering, Anhui University, Jiulong Road 111#, Hefei, Anhui 230601, China; School of Artificial Intelligence and Automation, Huazhong University of Science and Technology, Luoyu Road 1037#, Wuhan, Hubei 430070, China; Department of Orthopaedics, Institute of Orthopedics, Research Center for Translational Medicine, the Second Affiliated Hospital of Anhui Medical University, Furong Road 678#, Hefei, Anhui 230601, China; School of Internet, Anhui University, Jiulong Road 111# Hefei, Anhui 230601, China

**Keywords:** antimicrobial peptides, extreme multi label classification, ESM2, label clustering, hard negative sampling

## Abstract

In recent years, antimicrobial peptides (AMPs) have attracted interest as potential peptide antibiotic due to their broad-spectrum antibacterial activity and high target specificity. However, existing research on AMP prediction mainly focuses on their functional properties, such as antibacterial, antiviral, and anticancer. This emphasis has created a significant gap in identifying AMPs that specifically target pathogens. Given the large variety of pathogens and the sparsity and imbalance of labels, it is challenging to determine which specific pathogens AMPs can effective against. To address this issue, we present PepXML, a large language model-based tool for extreme multilabel classification of pathogen-targeted AMPs. Our first step involved constructing a benchmark dataset of AMPs and their corresponding targeted pathogens, sourced from public databases. In PepXML, the peptides are embedded using ESM2. Further, clustering on a specifically designed label co-occurrence graph and hard negative sampling were employed to address challenges on data sparsity and label imbalance. To validate the reliability of our predictive results, we conducted molecular docking studies focused on peptide-bilayer membrane interactions and performed molecular dynamics simulations to elucidate the mechanisms of peptide-pathogen interactions. We anticipate that PepXML will be a valuable resource for advancing peptide-based therapeutics. The data and Python codes of the PepXML model are available at https://github.com/YannanBin/PepXML.git.

## Introduction

The ongoing antibiotic resistance crisis has the potential to render antibiotics ineffective and increase the incidence of bacterial infections, exerting a profound threat to morbidity, mortality, and public health systems globally [1, 2]. The lack of new antibiotics has exacerbated this crisis, it is projected that by 2050, the global death toll from resistant infections could reach 10 million annually [3]. There is an urgent need to discover novel structural classes of antibiotics to address the ongoing antibiotic resistance crisis. In recent years, an increasing amount of research has focused on the development of peptide-based antibiotics [4–7]. Antimicrobial peptides (AMPs) represent a category of protein fragments or peptide segments possessing antimicrobial activity. With the broad-spectrum activity targeting a wide range of pathogens, AMPs offer a distinct advantage over conventional therapeutics that focus on specific pathogen species [8–11].

The methods employed to identify AMPs can be categorized into two main types. The first category encompasses wet laboratory techniques [12, 13], which require a lot of resources, time, and effort. To make these traditional wet lab approaches more efficient, researchers have adopted computational methods for high-throughput screening of AMPs [14–17]. These computational strategies have advanced significantly, evolving from simple sequence alignment algorithms and fuzzy logic models to more complex frameworks like traditional machine learning and deep learning algorithms. For instance, AmPEP [18], iAMP-CA2L [14], iAMP-Attenpred [15], iAMPCN [16], sAMPpred-GAT [19], and sAMP-VGG16 [20]. The ongoing improvement of these algorithms has led to better accuracy and faster processing in predictive models, making them more useful for identifying AMPs. With the development of artificial intelligence technologies, the types of prediction tasks in AMP research have expanded from simple binary classification to include multiclass and multilabel classifications (MLC) [14, 21–24]. For instance, Xiao *et al*. utilized an MLC approach to predict different subfunctions of AMPs, such as antibacterial, anticancer, antifungal, and antiviral activities [14]. In 2022, our research team introduced an MLC predictors termed PrMFTP, which employed deep neural network with a multihead self-attention mechanism to identify peptides across 21 distinct functional classes [22]. Subsequently, in 2023, we improve another MLC method called ETFC, which also employed deep learning along with a special loss function to classify peptides into the same 21 categories [21]. However, these MLC predictors were performed to identify the subfunctions of AMPs, there is still a significant gap in predicting which specific pathogens that AMPs can effectively against.

Current limitations in available data have led existing tools to primarily focus on a limited number of pathogens. For instants, Sharma *et al.* utilized ensemble learning and transfer learning to predict the minimum inhibitory concentration values against seven classes of ESKAPEE pathogens [25]. In the same year, Yao *et al.* developed a three-stage computational framework to predict AMPs and characterize their activity against 10 classes of pathogens, employing peptide features and a deep cascade forest algorithm [26]. However, the diversity of pathogens in nature extends far beyond these specified species. This underscores the urgent need for predictive models that can effectively identify pathogen- targeting AMPs across a broader range of species.

Unlike traditional MLC methods that typically handle five to 21 labels [21, 22, 24, 27], predicting pathogen targeting AMPs involves the management of hundreds or even thousands of labels. This task is precisely what extreme multilabel classification (XMLC) seeks to address, with pathogens acting as labels and AMPs as instances. In XMLC, the challenges of data sparsity and sample imbalance across various labels are more pronounced than in standard MLC. To address these issues, two main approaches have been employed: (i) label processing techniques, which encompass methods such as label clustering and label correlation analysis [28–30]; and (ii) data augmentation strategies, which aim to improve the representation of samples that are more prone to misprediction [31–33]. These methods have demonstrated varying degrees of success in mitigating the challenges associated with data sparsity and label sample imbalance in XMLC.

Predicting specific pathogen-targeting AMPs represents a typical XML task that faces challenges such as data sparsity and label imbalance. Addressing these issues is essential for the development of peptide-based therapeutics. In this work, we introduce PepXML, an XML model specifically designed for identifying specific pathogen-targeting AMPs. First, we create a comprehensive dataset by extracting information about AMPs and their corresponding targeted pathogens from public databases, thus forming an XML dataset. Next, we develop a baseline model utilizing large language model and deep learning techniques. To improve our predictions, we implement various strategies aimed at mitigating data sparsity and label imbalance. To validate the reliability and theoretical basis of our predictive results, we conduct molecular docking studies to investigate peptide-bilayer membrane interactions and perform molecular dynamics (MDs) simulations to elucidate the mechanisms underlying peptide-pathogen interactions. We anticipate that PepXML will be a valuable resource for advancing the field of peptide-based therapeutics and will provide insights into the development of targeted treatments for multidrug-resistant infections.

## Materials and methods

### Overall framework of PepXML

**Figure 1:**
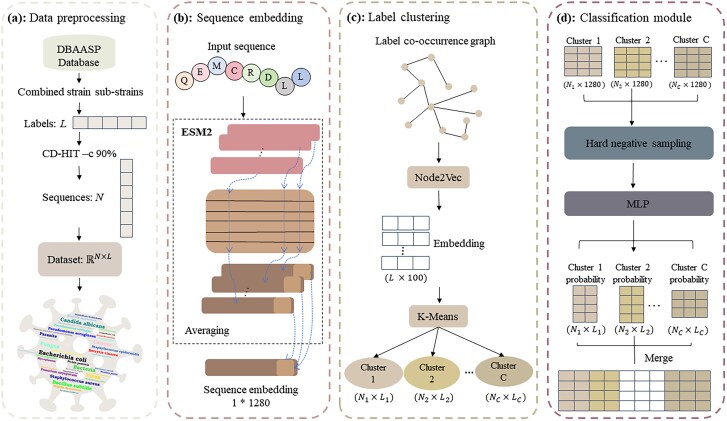
The overall framework of PepXML. PepXML consists of four distinct stages: data preprocessing (a), sequence embedding (b), label clustering (c), and classification module (d).

The construction of the PepXML model, illustrated in Fig. 1, involves four distinct stages: data preprocessing (i), sequence embedding (ii), label clustering (iii), and classification module (iv). Initially, we extracted AMPs and their corresponding targeted pathogens from the DBAASP database [34]. Within this XMLC framework, AMPs are considered as instances, while the corresponding pathogens serve as labels. The baseline XMLC model was developed using ESM2 [35]. Subsequently, a label co-occurrence graph is constructed, embedded using Node2Vec [36] and clustered labels into *C* clusters using K-Means. A multilayer perceptron (MLP) was subsequently employed to identify pathogen targeting AMPs within each cluster. The probabilities from different clusters integrate into the final results.

### Data preprocessing

The validated AMPs and their corresponding targeted pathogens were collected from the DBAASP v3 database [34], which contains peptide sequences and their corresponding targeted pathogens. In the original dataset, 18 352 AMPs, 6667 pathogens, and 137 718 AMP-pathogen associations were obtained (Table 1). The data processing involved three key steps (Supplementary Fig. S1): pathogen species processing, AMP sequence processing, and formation of AMP-pathogen matrix.

**Table 1:** Numbers of AMPs and pathogens in the datasets

**Item**	**Original dataset**	**Benchmark dataset**	**Independent test set** ^a^
AMP/Instance (*N*)	18 352	8444	586
Pathogen/Label (*L*)	6667	854	344
AMP-pathogen association	137 718	44 308	3926
Average number of labels per peptide	7.5	5.3	6.7
Average number of peptides per label	20.7	51.9	11.4

^a^Independent test set is selected from the CAMP_R4_ database, deleting the data exist in benchmark dataset.

#### Pathogen species processing

In the dataset, some pathogens are represented by multiple subspecies or variants (e.g. *MERS-COV PsV* and *MERS-COV-D509G PsV*), which could increase data sparsity. To enhance data density and improve the predictive performance, we combined different subspecies into their species [37], ensuring that only the species-level were retained. Following this subspecies consolidation, the benchmark dataset included 15 153 AMPs, 972 pathogens, and 80 865 AMP-pathogen associations.

#### Antimicrobial peptide sequence processing

AMPs are typically short sequences ranging from five to 50 amino acids (AAs). To minimize noise in the dataset, we excluded peptides longer than 50 AAs, and the length distribution of AMPs is displayed in Supplementary Fig. S2. Then, we applied the CD-HIT method [38] to remove peptide sequences with more than 90% sequence identity: (i) Removing similar sequences enhances the diversity of peptide sequences and mitigates the risk of performance overestimation caused by homology bias. This process ultimately improves the model’s generalization ability. (ii) Eliminating sequences may result in the loss of important information, which can affect the model’s predictive performance. Therefore, rely on existing studies in peptide function prediction [15, 39, 40], we specifically opt to remove peptide sequences with over 90% similarity, striking a balance between reducing redundancy and preserving the representativeness of the peptides dataset. As shown in Supplementary Fig. S1, the application of CH-HIT for redundancy removal during Data processing resulted in a reduction of peptide counts from 15 513 to 8444, while the number of pathogen targets for these peptides decreased from 972 to 854. This suggests that the original dataset included a considerable amount of highly similar peptides. Notably, after redundancy removal, the remaining pathogen targets continue to exhibit a substantial level of diversity.

**Figure 2:**
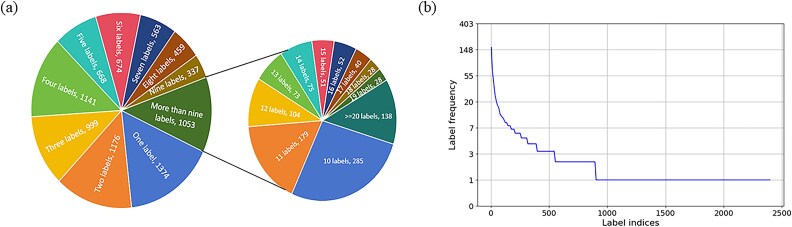
Distribution of AMPs across different label sets. (a) The distribution of AMPs across various label sets characterized by different quantities of labels. The numerical values depicted in the pie chart correspond to the total number of AMPs associated with each respective label set. (b) The distribution of AMPs across each individual label set.

The details of Benchmark dataset are shown in Table 1. Types of microbial and the number of pathogens in each type are shown in Supplementary Table S1. The distribution of AMPs across different label sets is illustrated in Fig. 2. The pie charts in Fig. 2a indicate that most AMPs can target multiple pathogens and have multiple labels. In contrast, the line graph in Fig. 2b shows that most label sets contain a limited number of AMPs, conforming to a power-law distribution. As shown in Table 1, the independent test set is selected from the CAMP_R4_ database [41], deleting the data exist in benchmark dataset. Furthermore, we apply four measures for label imbalance, including Imbalance ratio per label (IRLbl), Mean imbalance ratio (MeanIR), Maximum IRLbl (MaxIR), and Coefficient of variation of IRLbl (CVIR) [42]. The descriptions of these measures are exhibited in Supplementary Method S1.1. The IRLbl values of the benchmark dataset and independent test set are shown in Supplementary Fig. S3, and other measures of label imbalance on the benchmark dataset and independent test set are exhibited on Supplementary Table S2. The results indicate that both datasets are highly imbalanced and data sparse. Therefore, it is necessary to implement strategies to address these issues and enhance model performance. To address issues of sparse data and class imbalance in the dataset, we employed several strategies, including clustering and hard negative sampling (HNS).

#### Formation of antimicrobial peptide-pathogen matrix

These 44 308 AMP-pathogen associations were organized into an AMP-pathogen matrix ($M\in{\mathbb{R}}^{N\times L}$), which will be utilized for XMLC aimed at identifying pathogen targeting AMPs. In this matrix, *N* represents the number of AMPs (*N* = 8444), treated as instances for XMLC, while *L* denotes the number of pathogens (*L* = 854), considered as label for XMLC. Additionally, a value of 1 in the matrix indicates that the AMP exhibits resistance activity against the corresponding pathogen, while a value of 0 signifies the absence of such activity.

### Embedding module

A variety of deep learning models were employed to embed the peptide sequences, including traditional architectures and pretrained models [43, 44]. The traditional architectures include convolutional neural networks (CNNs) [45], long short-term memory (LSTM) [46], and their variants, including bidirectional LSTM (BiLSTM) [46], mLSTM [47], sLSTM [47], and extended LSTM (xLSTM) [47]. Detailed descriptions of each algorithm are provided in Supplementary Method S1.2. The pretrained models used in this study include the Transformer model [48], ESM2 [35], and Mamba [49].

#### ESM 2

ESM2 is a protein language model built on the Transformer architecture (Supplementary Fig. S4a), designed to learn rich sequence representations through self-supervised pretraining on extensive protein sequence datasets [35]. Compared to standard Transformer model, ESM2 offer advantages such as improved representation learning and adaptability to biological data [35]. Several studies have successfully utilized ESM2 to enhance predictive performance [50]. In this study, we utilized ESM2 to extract 1280-dimensional features from peptide sequences [51].

#### Mamba

Mamba incorporates a sliding window attention mechanism, known as the state-space models (SSMs) (Supplementary Fig. S4b), which is more computationally efficient than the standard Transformer architecture, making it particularly suitable for tasks involving long sequences [49]. As a state-of-the art pretrained model, Mamba utilizes the linear computational complexity to improve the efficiency of processing long sequences. Additionally, it incorporate a dynamic selection mechanism for key sequence features, which enhances its capability to effectively capture interactions among distant features within the sequence [49]. Here, we used Mamba to extract peptide sequence features, with the feature dimension being 768.

### Label clustering

In XMLC, complex co-occurrence relationships often exist among labels. To effectively represent these relationships, we construct an unweighted label co-occurrence graph in which each node corresponds to the label of an AMP, referred as the pathogen targeting AMPs. An undirected edge is established between different labels if they are associated with the same AMP. Subsequently, we employed Node2Vec [36] to embed each label using a random walk strategy that captures both local and global structures within the label graph. The embedding dimension was set to be 100, with a walk length of 5, and 100 random walks per node, resulting in a label embedding matrix $L\in{\mathbb{R}}^{LN\times 100}$, where *LN* denotes the number of labels (*LN* = 854). Finally, we applied different clustering methods to group the labels, thereby optimizing the predictive performance of the XMLC model. The clustering methods include K-Means [52], Fuzzy C-Means (FCM) [53], Gaussian mixture model (GMM) [54], and hierarchical clustering [55] techniques. Detailed descriptions of each method are provided in Supplementary Method S1.3.

### Classification module

An MLP classifier, combined with the baseline model (ESM2), is trained for each label cluster. Within each classifier module, HNS is used to enhance the model’s ability to capture hard negative samples. Ultimately, the prediction results from each cluster are merged. The subsequent sections will provide an introduction to the HNS strategy, the MLP classifier, and the merge module.

#### Hard negative sampling

In XMLC tasks, label sparsity and the high-dimensional attributes of the dataset hinder the model’s ability to learn information from samples and their corresponding labels effectively. To address these issues, we implement an HNS technique [32, 33]. This approach focuses on difficult negative samples, which are instances that are incorrectly predicted as positive with high confidence. We calculate hard negative samples independently for each label based on their confidence scores, thereby avoiding biases inherent in global sampling. This strategy enhances the predictive performance of the XMLC model. The detailed procedure is presented in Algorithm 1.

**Algorithm 1:** Hard negative sampling

**Input:** - Model: extreme multi-label classifier. - Data: training dataset. - Total_epoch = 100: total number of training epochs. - *E* = 1: number of epochs per sampling iteration. - *K* = 2: number of hard negative samples for each label. - Confidence_threshold = 0.5: confidence threshold for selecting hard negative samples. **Output:** ** Return** Index set of hard negative samples **Main:** 1. Training model 2. **While** epoch <= total_epoch: 3. **If** epoch % *E* == 0: 4. Probs = get model predictions (probability scores for each label). 5. Negative_mask = find negative samples (where true label is 0). 6. Negative_score = Probs ^*^ Negative_mask (calculate scores for negative samples, higher score means the negatives wrongly predicted with high confidence). 7. **If** Negative_score > Confidence_threshold: 8. Keep samples with Negative_score higher than the confidence threshold. 9. **Else**: 10. Print (‘No hard negative samples found!’) 11. Select hard negatives for each label: 12. Label_negative_number = get negative samples for this label. 13. **If** Label_negative_number > 0: 14. *K*_actual = min (*K*, Label_negative_number) ← select top *K* hard negative samples based on the highest confidence. 15. **End while**

#### Multilayer perceptron and merge module

The MLP classifier consists of two fully connected layers, batch normalization, activation functions, and a dropout layer. It maps the input dimension, which is the embedding dimension of peptide sequence, to the hidden layer dimension (256-dimension). A layer normalization layer is applied to standardize the output of the hidden layer with a ReLU activation function. Additionally, a dropout layer is utilized to prevent overfitting. Finally, a fully connected layer maps the hidden layer dimension to the number of output categories, which is the number of label types. Thus, the prediction results for each label cluster are obtained. Among these cluster (${N}_1\times{L}_1,{N}_2\times{L}_2,\dots, {N}_c\times{L}_c$), ${N}_1,{N}_2,\dots, {N}_c$ respectively represent the number of peptides in each cluster, and ${L}_1,{L}_2,\dots, {L}_c$ respectively represent the number of labels in each cluster. Finally, the prediction results of each label cluster are merged ($N\times L,L={L}_1+{L}_2+\dots +{L}_c$, *L* being the total number of labels).

### Evaluation metrics

In XMLC datasets, the label space is very large, but each instance only has a few relevant labels (see Table 1). Therefore, rank-based evaluation metrics have been widely used to compare XMLC models, including Precision at top *K* (P@K) and normalized Discounted Cumulative Gains at top *K* (nDCG@K) [56]. In this work, *K* was set to 1, 3, and 5.

P@K serves as a ranking-based performance metric that evaluates the accuracy of the model in predicting the top *K* most probable labels (targeted pathogens) for a given peptide. The model performance is represented by a predicted score vector of the *l*-th peptide ${\hat{y}}_l\in{\mathbb{R}}^L$ and a ground truth label of the *l*-th peptide vector ${y}_l\in{\left\{0,1\right\}}^L$. The calculation of P@K is performed as follows:


$$ \mathrm{P}@\mathrm{K}=\frac{1}{k}\sum_{l\in{\mathit{\operatorname{rank}}}_k\left(\hat{y}\right)}{y}_l $$

nDCG@K is an additional ranking-based metric that not only assesses prediction accuracy but also incorporates a weighting mechanism for labels based on their positions within the predicted ranking. This metric is particularly advantageous for addressing long-tail distribution challenges, as it evaluates the model effectiveness in accurately predicting rare labels at higher ranks. The calculation of nDCG@K is as follows:


$$ {\displaystyle \begin{array}{c} nDCG@K=\frac{DCG@K}{IDCG@K}=\frac{\sum_{l\in{\mathit{\operatorname{rank}}}_k\left(\hat{y}\right)}\frac{y_l}{\log \left(l+1\right)}}{\sum_{l=1}^{\min \left(k,{\left\Vert y\right\Vert}_0\right)}\frac{1}{\log \left(l+1\right)}}\end{array}} $$

In this work, higher values of P@K and nDCG@K indicate that the predictor demonstrates superior and more robust predictive performance. These two metrics serve as indicators of the model effectiveness in accurately identifying relevant labels, thereby reflecting its overall reliability in the given task.

Hamming loss (HL) is the common performance evaluation metrics in MLC, and measures the fraction of labels that are misclassified [57, 58]. A larger HL indicates a better performance. The calculation of HL is constructed as follows:


$$ HL=\frac{1}{p}{\sum_{i=1}^p}\frac{\mid h\left({x}_i\right)\triangle{Y}_i\mid }{L},p=N\times L $$

where *N* represents the number of peptides (instances), *L* is the number of pathogens (labels), $h\left({x}_i\right)$ represents the predicted value on the *i*-th label (0 or 1), while ${Y}_i$ represents the true value of the *i*-th label (0 or 1), $\triangle$ denotes the symmetric difference of the two sets and responds to the Exclusive OR operation in Boolean logic.

In this work, several binary classification evaluation metrics are used to assess the performance of XMLC models [57, 58]. These metrics include:

- MacroAUC: this metric calculates the area under the receiver operating characteristic curve (AUC) value for each label category individually. The average of these AUC values is then computed to obtain the MacroAUC.
  MicroAUC: in this metric, all samples from different label categories are combined into a single large dataset. The overall AUC value is then calculated for this combined dataset.
  MacroF1: first, the F1 value is calculated for each label category. The average of these F1 value is then computed to derive the MacroF1.
  MicroF1: the metric sums counts of true positives (TPs), false positives (FPs), true negatives (TNs), and false negatives (FNs) across all label categories. The overall F1 value for the dataset is then calculated based on these totals.

The definitions of MacroF1 and MicroF1 are provided by the respective formulas:


$$ MacroF1=\frac{1}{L}\sum_{i=1}^L\frac{2\cdotp Precision\cdotp Recall}{Precision+ Recall} $$

$$ Precision=\frac{TP}{TP+ FP}, Recall=\frac{TP}{TP+ FN} $$

$$ Micro F1=\frac{2\cdotp Micro\ Precision\cdotp Micro\ Recall}{Micro\ Precision+ Micro\ Recall} $$

$$ MicroPrecision=\frac{\sum_i{TP}_i}{\sum_i\left({TP}_i+{FP}_i\right)}, MicroRecall=\frac{\sum_i{TP}_i}{\sum_i\left({TP}_i+{FN}_i\right)} $$

### Model details

In this work, we utilized Python (version 3.8.19) and PyTorch (version 2.4.1) to construct the predictive model. The construction of the PepXML model was conducted on a computer equipped with an NVIDIA RTX 4090 GPU. To ensure the reproducibility of our experiments, we meticulously documented the software environment and the specific versions of all dependencies utilizing CUDA 12.4 and the corresponding drivers. These hyperparameters were optimized using grid search with five-fold cross-validation (5CV) on the benchmark dataset. Finally, the model was constructed with epochs = 100 (ensuring sufficient model learning), batch size = 32 (balancing memory usage and training stability), and learning rate = 0.001 (optimized using the Adam optimizer). Furthermore, the model architecture consisted of a MLP with a hidden layer size of 256 and a dropout rate of 0.3 to mitigate the risk of overfitting. For loss computation, we utilized Binary Cross-Entropy with Logits Loss (BCEWithLogitsLoss) in conjunction with the Sigmoid activation function to effectively address the MLC task. On the independent test set, we aimed to eliminate the effects of random initialization in the deep learning framework by training all models five times. The average scores from these repetitions were used as the final predicted results. We quantified the statistical significance of the differences between different the various methods using the t-test.

## Results and discussions

### Construction of the PepXML model

#### Performance comparison of various deep learning models

**Figure 3:**
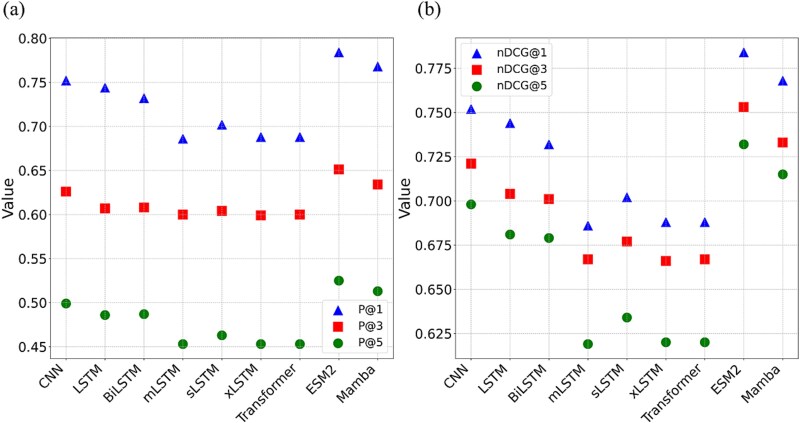
Performance comparison of XMLC models based on various deep learning algorithms with 5CV on benchmark dataset. (a) Evaluation metric of P@K. (b) Evaluation metric of nDCG@K.

In this work, we employed various embedding modules, including CNN, LSTMs (LSTM, BiLSTM, mLSTM, sLSTM, and xLSTM), Transformer, and large language models, to embed the peptide sequences. We systematically evaluated the performance of these models based on P@K and nDCG@K, which are the most important metrics among these evaluation metrics listed in Method section [33, 56]. As shown in Fig. 3, when applying 5CV on the benchmark dataset, we observed the performance across different deep learning models, as measured by P@K and nDCG@K. The results exhibit that ESM2 exhibits the highest values for both P@K and nDCG@K, while Mamba performs suboptimally. The results suggest that the residue information and their spatial positions extracted using ESM2 play a critical role on targeted pathogen identification for AMPs. This result aligns with the successful AMP predictions that utilize ESM2 feature extraction methods [51, 59, 60]. In addition, Mamba is a protein language model that represents a novel class of structured SSMs. It effectively captures long-range sequential dependency with subquadratic complexity [49]. However, the benchmark dataset shows that most AMPs are <20 amino acids in length (Supplementary Fig. S2). This limitation reduces Mamba’s ability to full utilize its long-range modeling capabilities. Moreover, Mamba’s sequence modeling approach focuses on global smoothness, which may compromise its capacity to capture essential residue-level features needed for accurate pathogen identification. Similarly, while LSTM and their variants are adept at capturing long-term dependencies in data [46, 47], the shorter lengths of AMPs diminish the advantages that these models typically provide. Furthermore, these LSTM models do not perform as well as the CNN model, which focus on local feature extraction [45].

Table 2 presents a performance comparison of XMLC models based on various deep learning algorithms on the independent test set. The results are consistent with those obtained from the benchmark dataset. Notably, the ESM2 model demonstrates the highest performance among all the XMLC models evaluated. Therefore, we selected the ESM2 model, which demonstrated the best overall performance, to develop the XML model for identifying pathogen targeting AMPs.

**Table 2:** Performance comparison of XMLC models based on various deep learning algorithms on the independent test set

**Model**	**P@K**			**nDCG@K**		
	**P@1**	**P@3**	**P@5**	**nDCG@1**	**nDCG@3**	**nDCG@5**
CNN	0.627^a^	0.579	0.509	0.627^a^	0.602^a^	0.554
LSTM	0.612^a^	0.558^a^	0.467^a^	0.612^a^	0.576^a^	0.512^a^
BiLSTM	0.623^a^	0.556^a^	0.492^a^	0.623^a^	0.583^a^	0.541^a^
mLSTM	0.590^a^	0.569^a^	0.386^a^	0.590^a^	0.575^a^	0.447^a^
sLSTM	0.597^a^	0.531^a^	0.477^a^	0.597^a^	0.556^a^	0.521^a^
xLSTM	0.615^a^	0.599	0.390^a^	0.615^a^	0.604^a^	0.456^a^
Transformer	0.616^a^	0.599	0.392^a^	0.616^a^	0.604^a^	0.458^a^
Mamba	0.630^a^	0.568	0.507	0.630^a^	0.600^a^	0.554
ESM2	0.653	0.589	0.515	0.653	0.618	0.567

^a^There is significant difference between ESM2 and other deep learning models with *P* < .05 (t-test).

#### Performance comparison of clustering algorithms

To address the issues of sparse data and class imbalance within the benchmark dataset, various clustering algorithms were employed in the baseline model (ESM2), including K-Means [52], FCM [53], GMM [54], and hierarchical clustering [55] algorithms. The performance comparison of these four algorithms with 5CV is presented in Table 3. The application of clustering algorithms enhances the performance of the baseline ESM2 model. The clustering method can effectively mitigate label redundancy and bolsters the model discriminative capability. Among these algorithms, the K-Means clustering method yielded the highest performance on the metrics of P@K and nDCG@K. Each algorithm resulted in a different number of clusters, and the corresponding label distributions were illustrated in Supplementary Fig. S5. The advantages of the K-Means method can be summarized in four key aspects: (i) K-Means demonstrates excellent performance in handling dense continuous features, efficiently partitioning pathogen labels into semantically similar sub clusters [61]. (ii) Node2Vec is used to embed the pathogen label co-occurrence graph, generating dense representation vectors for each label in a high-dimensional space. The label vectors exhibit favorable Euclidean geometric properties, making their spatial structure more suitable for clustering with K-Means [62]. (iii) As shown in Supplementary Fig. S4, visualizations of label clusters from various clustering methods reveals that K-Means produces a relatively balanced distribution of label quantities across clusters. This helps avoid the small clusters or redundant clusters that occur in FCM, GMM, and hierarchical methods. (iv) K-Means demonstrates rapid convergence to a local optimum when initialized with reasonable cluster centers and offers flexibility in adjusting the number of clusters.

**Table 3:** Performance comparison of the baseline model with different clustering algorithms on benchmark dataset

**Algorithm**	**P@K**			**nDCG@K**		
	**P@1**	**P@3**	**P@5**	**nDCG@1**	**nDCG@3**	**nDCG@5**
Baseline model^a^	0.784	0.651	0.525	0.784	0.753	0.732
K-Means (10)^b^	**0.859**	**0.712**	**0.583**	**0.859**	**0.840**	**0.828**
FCM (2)	0.815	0.674	0.548	0.815	0.788	0.772
Hierarchical (3)	0.798	0.661	0.533	0.798	0.769	0.749
GMM (7)	0.843	0.690	0.559	0.843	0.811	0.794

^a^The ESM2 model is regarded as baseline model.

^b^The number in parentheses is the count of clusters obtained using the corresponding clustering algorithms.

**Figure 4:**
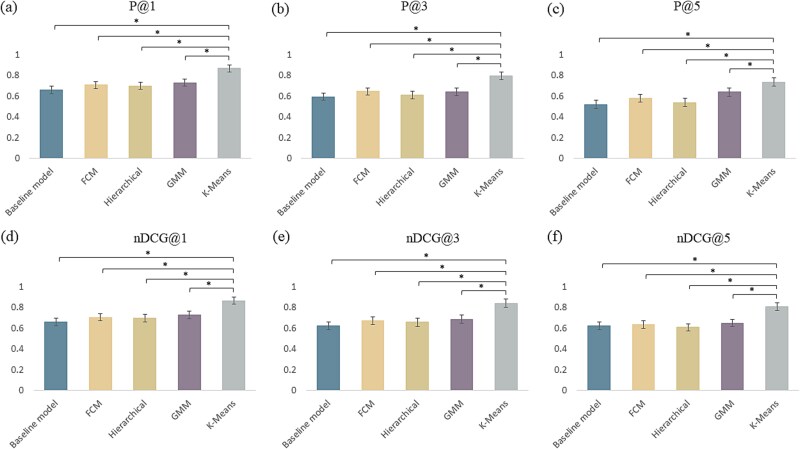
Performance comparison of various clustering methods on the independent test set. *There is significant difference between K-Means and other methods with *P* < .05 (t-test). The ESM2 model was treated as the baseline model.

Fig. 4 illustrates a performance comparison of the baseline model (ESM2) using different clustering methods on the independent test set. The results align with those obtained from 5CV conducted on the benchmark dataset. Importantly, K-Means exhibits the best performance among the three clustering methods evaluated. Consequently, we selected K-Means as the preferred clustering algorithm for the development of the XMLC model aimed at identifying pathogen targeting AMPs.

#### Comparative study of data sparsity mitigation strategies

To further mitigate the effects of data sparsity on model performance, we implemented various strategies, including interaction-attention (IA) [30], HNS [32, 33], and label interaction learning (LIL) [56] strategies. The IA strategy facilitates the mapping of AMPs and their targeting pathogens into a share latent semantic space, thereby addressing data sparsity issues. The detailed description of IA is provided in Supplementary Method S1.3. The HNS strategy emphasizes the incorrectly predicted negative samples to extract more relevant features associated with negative samples. Meanwhile, the LIL strategy leverages label correlations to assess the importance of various labels and adaptively adjusts label predictions based on their correlations. The detailed descriptions of IA and LIL were shown in Supplementary Method S1.4 and S1.5, respectively.

As illustrated in Table 4, HNS outperforms K-Means on the performance improvement of the baseline model. The HNS strategy, which concentrated on utilizing incorrect negative samples, significantly improved the extraction of information from these negative samples. Additionally, K-Means clustering was employed to tackle the challenges of sparse data and class imbalance present in the benchmark dataset. Considering the positive contributions of the K-Means and HNS strategies on performance improvement, we combined these two strategies. As shown in Supplementary Fig. S6, the K-Means+HNS model exhibits a notable enhancement in prediction accuracy, and outperforms the K-Means and HNS models. Table 4 exhibits that the IA and LIL strategies do not significantly affect model performance. Furthermore, we explored the combination of IA or LIL with other strategies, however, this also did not yield any performance improvements (data not shown).

**Table 4:** Performance comparison of different data sparsity mitigation strategies with 5CV on benchmark dataset

**Strategy**	**P@K**			**nDCG@K**		
	**P@1**	**P@3**	**P@5**	**nDCG@1**	**nDCG@3**	**nDCG@5**
Baseline model^a^	0.784	0.651	0.525	0.784	0.753	0.732
K-Means	0.859	0.712	0.583	0.859	0.840	0.828
IA^b^	0.781	0.651	0.523	0.781	0.750	0.729
HNS^c^	0.874	0.746	0.619	0.874	0.867	0.859
LIL^d^	0.785	0.651	0.526	0.785	0.756	0.736
K-Means + HNS (PepXML)	**0.931** ^**e**^	**0.794**	**0.666**	**0.931**	**0.934**	**0.932**

^a^The ESM2 model is regarded as baseline model.

^b^IA represents Interaction-Attention.

^c^HNS represents hard negative sampling.

^d^LIL represents label interaction learning.

^e^The highest value is highlighted in bold.

**Figure 5:**
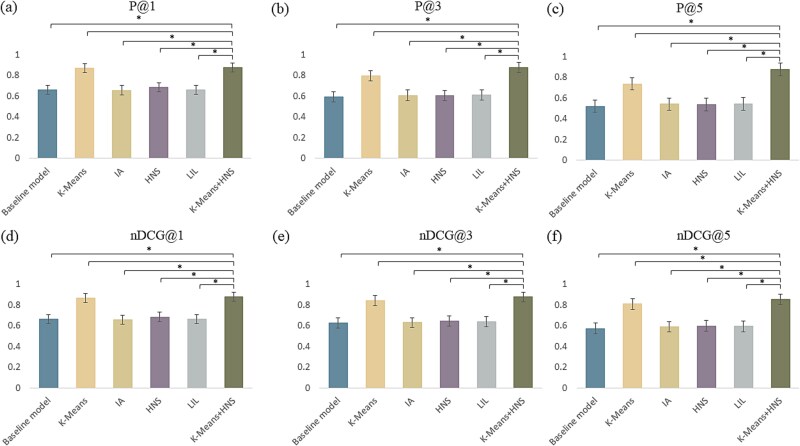
Performance comparison of various data sparsity mitigation strategies on the independent test set. * There is significant difference between the K-Means+HNS strategies and other strategies with *P* < .05 (t-test). The ESM2 model was treated as the baseline model.

Fig. 5 provides a performance comparison of the baseline model (ESM2) utilizing various data sparsity mitigation strategies on the independent test set. The results are generally consistent with those observed on the benchmark dataset. However, the HNS strategy shows reduced performance on the test set compared to the benchmark dataset, indicating that the HNS strategy has encountered overfitting issues with the benchmark dataset. However, when HNS is combined with K-Means, it effectively addresses the overfitting problem and improves the model’s overall performance. Consequently, we utilized ESM2 as the baseline model integrating K-Means and HNS to develop the PepXML model for the identification of pathogen targeting AMPs.

### Parameter optimization

Within the HNS module, there are two hyperparameters were optimized, including the number of HNSs per label (*K*) and the number of sampling epochs (*E*). Notably, when *K* is set to 0, it is the PepXML model without HNS. As presented in Table 5, the results demonstrate that increasing *K* from 0 to 1 results in a significant improvement in the P@1 metric, rising from 0.859 to 0.937. This finding suggests that the incorporation of hard negative samples is crucial for enhancing the model’s discriminative ability. However, as *K* increases further (*K* = 2–4), the metrics exhibit only slight fluctuations and tend to plateau or decline slightly. This decline may occur due to an excessive number of negative samples, which can introduce misclassification noise and adversely affect the model’s convergence direction. Therefore, we determined that *K* = 1 is the most advantageous choice. Upon fixing *K* at 1, we examined the impact of varying *E* across the values of [3, 5, 10]. The results indicate that the change of *E* has limited effect on the model’s performance. Consequently, we propose *K* = 1 and *E* = 10 as the optimal configuration, as this choice effectively balances performance and computational efficiency.

**Table 5:** Parameter optimization of *K* and *E* used in the NHS strategies with 5CV on benchmark dataset

**Evaluation**	***K*** ^**a**^ **(*E* = 10)**					***E*** ^**b**^ **(*K* = 1)**		
	***K = 0***	***K = 1***	***K = 2***	***K = 3***	***K = 4***	***E = 3***	***E = 5***	***E = 10***
P@1	0.859	**0.937** ^c^	0.931	0.934	0.932	0.934	0.933	**0.937**
P@3	0.712	**0.796**	0.794	0.795	0.794	0.795	0.794	**0.796**
P@5	0.583	**0.667**	0.666	**0.667**	**0.667**	**0.667**	0.665	**0.667**
nDCG@1	0.859	**0.937**	0.931	0.934	0.932	0.934	0.933	**0.937**
nDCG@3	0.840	**0.937**	0.934	0.935	0.935	0.936	0.935	**0.937**
nDCG@5	0.828	**0.935**	0.932	0.934	0.934	0.934	0.932	**0.935**

^a^ *K* represents the number of hard negative samples for each label.

^b^ *E* represents the number of sampling epochs.

^c^The highest value is highlighted in bold.

### Ablation experiments

To further explore the significance of ESM2, K-Means, and HNS within PepXML, we conducted ablation experiments to elucidate the contributions of these components. Additionally, we compared PepXML with the following variants to assess their relative impacts on model performance:

- w/o ESM2 refers to a variant that does not employ ESM2, instead utilizing a CNN in its place.
  w/o K-Means refers to a variant that does not employ any clustering algorithm.
  w/o HNS refers to a variant that does not employ HNS.

Table 6 exhibits a performance comparison of PepXML and its various variants on the independent test set. The results illustrate that each module is essential for overall performance of PepXML. Notably, the variants of the w/o K-Means model exhibited the most significant decline in performance, followed by the variants of the w/o ESM2 and w/o HNS models. These findings suggest that the K-Means strategy is the most pivotal component of PepXML, highlighting the considerable impact that addressing data sparsity issues has on improving model performance.

**Table 6:** Performance comparison of PepXML and its various variants on the independent test set

Evaluation	PepXML	w/o ESM2^a^	w/o K-Means^b^	w/o HNS^c^
P@1	**0.877** ^d^	0.865^e^	0.685^e^	0.868
P@3	0.806	**0.807**	0.607^e^	0.796
P@5	**0.748**	0.728	0.539^e^	0.737
nDCG@1	**0.877**	0.865^e^	0.685^e^	0.868
nDCG@3	**0.854**	0.852	0.644^e^	0.843
nDCG@5	**0.821**	0.803^e^	0.597^e^	0.809^e^
HL	**0.004**	0.005^e^	0.007^e^	**0.004**
MacroAUC	**0.976**	0.968^e^	0.874^e^	0.969^e^
MicroAUC	**0.993**	0.990^e^	0.950^e^	0.991^e^
MacroF1	**0.639**	0.327^e^	0.526^e^	0.598
MicroF1	**0.722**	0.575^e^	0.528^e^	0.680

^a^w/o ESM2 represents the ESM2 model replacing by CNN.

^b^w/o K-Means indicates the model without clustering module.

^c^w/o HNS indicates the model without HNS. ^d^The best value is highlighted in bold.

^e^means that there is significant difference between the PepXML model and its variants with *P* < .05 (t-test).

### Performance comparison of PepXML with existing methods

To date, there are few computational methods for predicting pathogen-targeted peptides/AMPs, and the existing methods primarily focus on a limited number of pathogens. For instance, ESKAPEE-MICpred was designed to target seven pathogens [25], while AMPActiPred targeted 10 pathogens [26]. The limited scope of these methods, combined with their reliance on binary classification techniques, makes comparison with the proposed PepXML model impractical (PepXML targeted 854 pathogens). Moreover, there are currently no XMLC methods specifically related to peptides, and existing text-based XMLC approaches do not incorporate peptide feature extraction, rendering them unsuitable for comparison. Since PepXML is based on XMLC and fits within the category of MLC, we compared PepXML and its baseline method (ESM2) with existing peptide MLC methods, including PrMFTP (21 labels, utilizing a CNN-BiLSTM architecture) [22], TransImbAMP (6 labels, employing a Transformer framework) [63], and ETFC (21 labels, integrating TextCNN with a multilabel focal dice loss function) [21]. The detailed information of hyperparameters for these methods is shown in Supplementary Table S3. The baseline model (ESM2) is a standard MLC approach that does not employ XMLC strategies.

To ensure the reproducibility and reliability of the results, detailed information of hyperparameter for each method is provided in Supplementary Table S2. All methods were evaluated on the independent test set using several performance metrics, including P@K, nDCG@K, HL, MacroAUC, MicroAUC, MacroF1, and MicroF1. Table 7 shows that PepXML significantly outperforms the baseline model (ESM2), PrMFTP, TransImbAMP, and ETFC across all metrics. These results indicate that the integration of K-Means and HNS into the baseline model (ESM2) leads to a substantial improvement in performance. Furthermore, the baseline model (ESM2) demonstrates superior capability in peptide feature extraction compared to other deep learning algorithms.

**Table 7:** Performance comparison of PepXML with other MLC methods on the independent test set

**Evaluation**	**PepXML**	**ESM2**	**PrMFTP**	**TransImbAMP**	**ETFC**
P@1	**0.877** ^a^	0.661^b^	0564^b^	0.620^b^	0.602^b^
P@3	**0.806**	0.595^b^	0.513^b^	0.578^b^	0.551^b^
P@5	**0.748**	0.521^b^	0.407^b^	0.448^b^	0.480^b^
nDCG@1	**0.877**	0.661^b^	0.564^b^	0.620^b^	0.602^b^
nDCG@3	**0.854**	0.625^b^	0.531^b^	0.586^b^	0.567^b^
nDCG@5	**0.821**	0.574^b^	0.458^b^	0.488^b^	0.519^b^
HL	**0.004**	0.007^b^	0.008^b^	0.007^b^	0.008^b^
MacroAUC	**0.976**	0.856^b^	0.725^b^	0.813^b^	0.657^b^
MicroAUC	**0.993**	0.943^b^	0.884^b^	0.919^b^	0.866^b^
MacroF1	**0.639**	0.490^b^	0.089^b^	0.010^b^	0.185^b^
MicroF1	**0.722**	0.499^b^	0.297^b^	0.327^b^	0.485^b^

^a^The best value is highlighted in bold.

^b^means that there is significant difference between the PepXML model and existing methods with *P* < .05 (t-test).

We utilized the Python package *thop* (https://github.com/Lyken17/pytorch-OpCounter) to calculate the time complexity (measured in floating point operations per second, FLOPs) and space complexity (defined as the number of parameters in the model, Parameters) of PepXML. We then compared these metrics with various MLC models. Supplementary Table S4 indicates that both FLOPs and Parameters of both PepXML and the baseline model (ESM2) are greater than those of existing methods. While using ESM2 as the baseline model enhances the performance of PepXML, it also increases model’s complexity.

### Case study

A performance comparison of various existing MLC methods demonstrated that PepXML was superior to other methods in the XMLC of pathogen targeting AMPs. Research indicates that AMPs inhibit and kill bacteria by disrupting the bacterial cell membrane [8, 9], we conducted peptide-membrane docking (PMD) [64] and MDs simulation [65, 66] to validate the interaction of peptide-pathogen and support the accuracy of the predicted results of the PepXML model.

The case study involved an affinity analysis of AMPs with the membranes of both Gram-positive bacteria and Gram-negative bacteria, utilizing PMD and MD simulations. Initially, PEP-FOLD3 [67] was utilized to generate the 3D structure of the peptide. We constructed the membrane lipid composition ratios of Gram-positive bacteria and Gram-negative bacteria [64] utilizing CHARMM-GUI (Supplementary Table S5) [68]. The lipid composition and their respective ratios were meticulously designed to reflect real biological membranes, thereby ensuring physiological relevance of the simulations. Following the construction of the systems, we performed energy minimization and equilibration using GROMACS [69], which was succeeded by a 500 ns production run at a temperature of 310 K. The CHARMM36 force field and the TIP3P water model were employed, with the addition of Na^+^/Cl^−^ ions to achieve system neutrality. During the simulation, system coordinates were recorded every 100 ns to facilitate further analysis. Finally, we analyzed the simulation trajectories using Visual Molecular Dynamics (VMDs) [70] and GROMACS.

**Figure 6:**
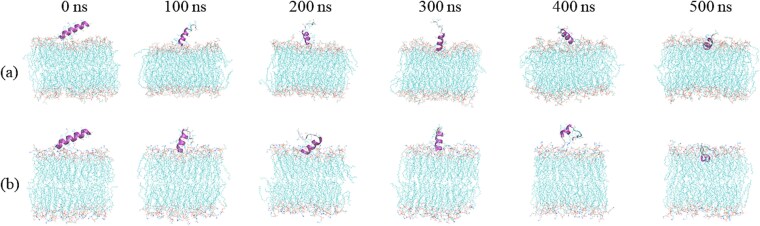
VMD visualization for the PMD of Pep1 (KKSFFKKLTSVASSVLS) interacting with bacterial bilayer membrane. (a) *S. aureus* and (b) *E. coli*.

Table 8 presents the affinity of the predicted AMPs (from PepXML) to the bilayer membrane. The results indicate that the five peptides identified from the PepXML model possess significant membrane-binding capabilities. Specifically, Pep3 (ARLDVASEFRKKWNKWALSR) demonstrates a strong affinity for the membrane of *Escherichia coli*, whereas Pep2 (RWKIFKKIPKFLHSAKKF) exhibits a strong affinity for the membrane of *Staphylococcus aureus*. Among these peptides, Pep1 (KKSFFKKLTSVASSVLS) was selected to investigate its interactions with bilayer membrane of bacterial. For the Gram-positive bacteria, we focused on *S. aureus*, while *E. coli* served as the representative for Gram-negative bacteria. Pep1 was screened by PepXML, identifying the capability to target a diverse range of pathogens, specifically including *S. aureus* and *E. coli*. Fig. 6 illustrates a side-view analysis of the changes in peptide-membrane interactions over a simulation period ranging from 0 to 500 ns for both *S. aureus* and *E. coli*. As shown, it demonstrated by the initial presence of the peptide on the membrane surface, followed by its gradual embedding and deepening into the membrane interior with time varying. During the process, the bilayer membranes gradually collapse and disintegrate. These analyses offer valuable insights into the dynamic behavior and stability of peptide interactions with bacterial membranes. The simulation results indicate that the Pep1 is capable of interacting with the bilayer membranes of both *S. aureus* and *E. coli*.

**Table 8:** The affinity of the predicted peptides to the *E. Coli* and *S. Aureus* bilayer membrane

Ligands	Pep1^a^	Pep2	Pep3	Pep4	Pep5
***E. coli* membrane**					
Round1_△G	−3.697	−4.381	−4.664	−4.131	−3.628
Round2_△G	−4.417	−4.128	−4.379	−4.234	−4.058
Round3_△G	−4.413	−3.788	−4.677	−4.381	−3.561
Average_△G	−4.176	−4.099	−4.573	−4.249	−3.749
***S. aureus* membrane**					
Round1_△G	−4.477	−4.004	−4.083	−4.882	−3.860
Round2_△G	−3.865	−5.537	−4.465	−4.225	−3.960
Round3_△G	−4.975	−5.511	−4.474	−4.384	−3.860
Average_△G	−4.439	−5.017	−4.341	−4.497	−3.893

^a^Pep1: KKSFFKKLTSVASSVLS; Pep2: RWKIFKKIPKFLHSAKKF; Pep3: ARLDVASEFRKKWNKWALSR; Pep4: IGKKFKRIVQRIKKFLRNL; Pep5: ITKQITKQLNRQTKIQSK.

**Figure 7:**
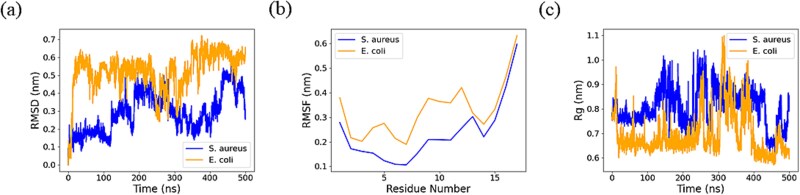
The corresponding evolution of key structural parameters for Pep1 (KKSFFKKLTSVASSVLS) interaction with *S. aureus* and *E. coli*. (a) RMSD, (b) RMSF, and (c) Rg.

Additionally, Fig. 7 presents the corresponding evolution of key structural parameters for the Pep1, including the Root Mean Square Deviation (RMSD), Root Mean Square Fluctuation (RMSF), and Radius of Gyration (Rg). According to the RMSD analysis, the fluctuation range of the peptide membrane system of *S. aureus* is relatively small, while the value in the *E. coli* peptide membrane system is significantly higher and fluctuates greatly, indicating that this peptide has higher stability in the membrane of *S. aureus*. The RMSF analysis indicates that the RMSF values of the residues in the *S. aureus* membrane-peptide system are all lower than those in the *E. coli* peptide membrane system. However, the RMSF values at both the N-terminal and C-terminal ends are relatively high, demonstrating greater flexibility. The results are consistent with the unfolding of the peptide structure observed in Fig. 6. Furthermore, the overall Rg in the *S. aureus* membrane-peptide system is relatively high, suggesting that the peptides exhibit a more compact and dynamic change in *S. aureus*, while they are relatively loose in *E. coli*. The results of PMD and MD simulation further demonstrate the accuracy and rationality of the PepXML prediction results. In further work, we will employ biological experiments to validate these *S. aureus*, *E. coli*, and other pathogens targeting AMPs. The approach will further support the predictive performance of our model and provide additional datasets of specific pathogens targeting AMPs.

## Conclusion

In this work, we introduced a novel method, termed PepXML, which employed an XMLC model that integrates ESM2 and K-Means clustering for the prediction of pathogen targeting AMPs. ESM2 serves as the foundation for our baseline model, while K-Means and HNS methods were applied to effectively address the challenges of data sparsity and label imbalance. The PepXML model is designed with a modular architecture, enabling parallel processing and easy expansion of its components. The first component is the data processing module, where each step has been standardized to ensure consistency and efficiency. The second component is the sequence embedding, which utilizes ESM2. This trained model is highly modular, allowing for integration with other deep learning modules. The third component is the label clustering model, which incorporates a label co-occurrence graph, Node2Vec embeddings, and K-Means clustering, all while maintaining standardization and modularity. Finally, the classification module serves as the core element of the predictive model, also demonstrating a high degree of modularity. We have made the code for the PepXML model available at https://github.com/YannanBin/PepXML.git. The Readme file provides detailed instructions for installation and usage.

Comparative analyses indicate that PepXML outperforms other MLC methods across all evaluation metrics. Additionally, the analysis of molecular docking studies and MD simulations elucidate the mechanisms underlying peptide-pathogen interactions. We anticipate that PepXML will become a valuable resource for advancing peptide-based therapeutics and will contribute to the development of targeted treatments for multidrug-resistant infections.

In this work, the PepXML model is introduced to predict the pathogen targeting AMPs, but there are some limitations for the development of AMP-based drugs, including issues related to high toxicity, lack of selectivity, insufficient stability, and potential immunogenicity of AMPs. Currently, methods for predicting the drug properties of AMPs are limited. This scarcity is primarily due to insufficient data, limited research on feature extraction methods for specific properties, and a lack of comprehensive prediction platforms. By overcoming these challenges, it is possible to predict various drug properties of peptides more accurately. In further work, we will introduce loss functions to tackle the long-tail distribution issues present in XML data. Additionally, we will explore to employ label weights and implement minority label sampling mechanisms to alleviate the impact of label sparsity on model performance. Moreover, PepXML is capable of predicting pathogen targeting for peptides with unknown functions that are not included in the dataset. However, the absence of specific pathogen labels in the dataset prevents accurate predictions for these pathogen-targeted peptides. To overcome this limitation, the dataset can be expanded by collecting additional data on pathogen-targeting peptides, thereby enhancing the ability to make predictions for a broader range of these peptides. Despite these challenges, AMPs remain a valuable resource in the fight against drug-resistant pathogens. Further work will focus on enhancing computational methods to better assess and mitigate these disadvantages related to toxicity, stability, and immunogenicity of AMPs.

**Key Points**

- We introduce PepXML, a novel extreme multilabel model designed to predict specific pathogens that AMPs can effectively against.
- PepXML integrates a large language model with label clustering and hard negative sampling strategies to enhance predictive performance.
- Label clustering and hard negative sampling effectively addresses issues of data sparsity and label imbalance, resulting in improved model performance.
- Peptide-bilayer membrane docking and molecular dynamics simulations are utilized to elucidate the mechanisms underlying peptide-pathogen interactions.

## Supplementary Material

PepXML-Supplementary_bbaf548

## Data Availability

The data and Python code used in the PepXML model are available at https://github.com/YannanBin/PepXML.git.

## References

[1] Ferri M, Ranucci E, Romagnoli P. et al. Antimicrobial resistance: a global emerging threat to public health systems. Crit Rev Food Sci 2017;57:2857–76. 10.1080/10408398.2015.1077192. 26464037

[2] Salam MA, al-Amin MY, Salam MT. et al. Antimicrobial resistance: a growing serious threat for global public health. Health 2023;11:1946. 10.3390/healthcare11131946. PMC10340576 37444780

[3] Wong F, Zheng EJ, Valeri JA. et al. Discovery of a structural class of antibiotics with explainable deep learning. Nature 2023;626:177–85. 10.1038/s41586-023-06887-8. 38123686 PMC10866013

[4] Lei J, Sun L, Huang S. et al. The antimicrobial peptides and their potential clinical applications. Am J Transl Res 2019;11:3919–31. 31396309 PMC6684887

[5] Zhu Y, Hao W, Wang X. et al. Antimicrobial peptides, conventional antibiotics, and their synergistic utility for the treatment of drug-resistant infections. Med Res Rev 2022;42:1377–422. 10.1002/med.21879. 34984699

[6] Xuan J, Feng W, Wang J. et al. Antimicrobial peptides for combating drug-resistant bacterial infections. Drug Resist Updat 2023;68:100954. 10.1016/j.drup.2023.100954. 36905712

[7] Kim D-I, Han SH, Park H. et al. Pseudo-isolated α-helix platform for the recognition of deep and narrow targets. J Am Chem Soc 2022;144:15519–28. 10.1021/jacs.2c03858. 35972994

[8] Huy Xuan L, Tung Truong T, Tuan Hiep T. Antimicrobial peptides-advances in development of therapeutic applications. Life Sci 2020;260:118407. 10.1016/j.lfs.2020.118407. 32931796 PMC7486823

[9] Muttenthaler M, King GF, Adams DJ. et al. Trends in peptide drug discovery. Nat Rev Drug Discov 2021;20:309–25. 10.1038/s41573-020-00135-8. 33536635

[10] Pereira AJ, de Campos LJ, Xing H. et al. Peptide-based therapeutics: challenges and solutions. Med Chem Res 2024;33:1275–80. 10.1007/s00044-024-03269-1.

[11] Thakur A, Alajangi HK, Sharma A. et al. Stigmurin encapsulated PLA–PEG ameliorates its therapeutic potential, antimicrobial and antiproliferative activities. Discover Nano 2025;20:50. 10.1186/s11671-025-04224-8. 40063147 PMC11893973

[12] Luo XY, Hu CM, Yin Q. et al. Dual-mechanism peptide SR25 has broad antimicrobial activity and potential application for healing bacteria-infected diabetic wounds. Adv Sci 2024;11:e2401793. 10.1002/advs.202401793. PMC11321617 38874469

[13] Xiao B, Liao X, Wang H. et al. BigPEN, an antimicrobial peptide of penaeidin family from shrimp Litopenaeus vannamei with membrane permeable and DNA binding activity. Fish Shellfish Immun Rep 2021;2:100034–4. 10.1016/j.fsirep.2021.100034. PMC9680095 36420505

[14] Xiao X, Shao YT, Cheng X. et al. iAMP-CA2L: a new CNN-BiLSTM-SVM classifier based on cellular automata image for dentifying antimicrobial peptides and their functional types. Brief Bioinform 2021;22:bbab209. 10.1093/bib/bbab209. 34086856

[15] Xing W, Zhang J, Li C. et al. iAMP-Attenpred: a novel antimicrobial peptide predictor based on BERT feature extraction method and CNN-BiLSTM-attention combination model. Brief Bioinform 2023;25:bbad443. 10.1093/bib/bbad443. 38055840 PMC10699745

[16] Xu J, Li F, Li C. et al. iAMPCN: a deep-learning approach for identifying antimicrobial peptides and their functional activities. Brief Bioinform 2023;24:bbad240. 10.1093/bib/bbad240. 37369638 PMC10359087

[17] Chamoli T, Khera A, Sharma A. et al. Peptide utility (PU) search server: a new tool for peptide sequence search from multiple databases. Heliyon 2022;8:e12283. 10.1016/j.heliyon.2022.e12283. 36590540 PMC9800339

[18] Bhadra P, Yan J, Li J. et al. AmPEP: sequence-based prediction of antimicrobial peptides using distribution patterns of amino acid properties and random forest. Sci Rep 2018;8:1697. 10.1038/s41598-018-19752-w. 29374199 PMC5785966

[19] Yan K, Lv H, Guo Y. et al. sAMPpred-GAT: prediction of antimicrobial peptide by graph attention network and predicted peptide structure. Bioinformatics 2023;39:btac715. 10.1093/bioinformatics/btac715. 36342186 PMC9805557

[20] Pandey P, Srivastava A. sAMP-VGG16: force-field assisted image-based deep neural network prediction model for short antimicrobial peptides. Proteins 2025;93:372–83. 10.1002/prot.26681. 38520179

[21] Fan H, Yan W, Wang L. et al. Deep learning-based multi-functional therapeutic peptides prediction with a multi-label focal dice loss function. Bioinformatics 2023;39:btad334. 10.1093/bioinformatics/btad334. 37216900 PMC10234765

[22] Yan W, Tang W, Wang L. et al. PrMFTP: multi-functional therapeutic peptides prediction based on multi-head self-attention mechanism and class weight optimization. PLoS Comput Biol 2022;18:e1010511. 10.1371/journal.pcbi.1010511. 36094961 PMC9499272

[23] Cao R, Hu W, Wei P. et al. FFMAVP: a new classifier based on feature fusion and multitask learning for identifying antiviral peptides and their subclasses. Brief Bioinform 2023;24:bbad353. 10.1093/bib/bbad353. 37861174

[24] Tang W, Dai R, Yan W. et al. Identifying multi-functional bioactive peptide functions using multi-label deep learning. Brief Bioinform 2022;23:bbab414. 10.1093/bib/bbab414. 34651655

[25] Sharma R, Shrivastava S, Singh SK. et al. Artificial intelligence-based model for predicting the minimum inhibitory concentration of antibacterial peptides against ESKAPEE pathogens. IEEE J Biomed Health 2024;28:1949–58. 10.1109/JBHI.2023.3271611. 37115837

[26] Yao L, Guan J, Xie P. et al. AMPActiPred: a three-stage framework for predicting antibacterial peptides and activity levels with deep forest. Protein Sci 2024;33:e5006. 10.1002/pro.5006. 38723168 PMC11081525

[27] Yan K, Lv H, Shao J. et al. TPpred-SC: multi-functional therapeutic peptide prediction based on multi-label supervised contrastive learning. SCIENCE CHINA Inf Sci 2024;67:212105:1–12. 10.1007/s11432-024-4147-8.

[28] Zong D, Sun S. BGNN-xml: bilateral graph neural networks for extreme multi-label text classification. IEEE T Knowl Data En 2023;35:6698–709.

[29] Kharbanda S, Gupta D, Schultheis E. et al. Gandalf: Learning label-label correlations in extreme multi-label classification via label features. ACM SIGKDD 2024;KDD'24:1360–371. 10.1145/3637528.3672063.

[30] Huang X, Chen B, Xiao L. et al. Label-aware document representation via hybrid attention for extreme multi-label text classification. Neural Process Lett 2022;54:3601–17. 10.1007/s11063-021-10444-7.

[31] Zhan J, Mao J, Liu Y. et al. Optimizing dense retrieval model training with hard negatives. ACM SIGIR 2021;SIGIR'21:1503–12. 10.1145/3404835.3462880.

[32] Qaraei M, Babbar R. Meta-classifier free negative sampling for extreme multilabel classification. Mach Learn 2024;113:675–97. 10.1007/s10994-023-06468-w.

[33] Dahiya K, Saini D, Mittal A. et al. Deepxml: A deep extreme multi-label learning framework applied to short text documents. ACM WSDM 2021;WSDM'21:31–9. 10.1145/3437963.3441810.

[34] Pirtskhalava M, Amstrong AA, Grigolava M. et al. DBAASP v3: database of antimicrobial/cytotoxic activity and structure of peptides as a resource for development of new therapeutics. Nucleic Acids Res 2021;49:D288–97. 10.1093/nar/gkaa991. 33151284 PMC7778994

[35] Du Z, Ding X, Xu Y. et al. UniDL4BioPep: a universal deep learning architecture for binary classification in peptide bioactivity. Brief Bioinform 2023;24:bbad135. 10.1093/bib/bbad135. 37020337

[36] Grover A, Leskovec J, M. Assoc Comp. Node2Vec: Scalable feature learning for networks. ACM SIGKDD 2016;KDD'16:855–64. 10.1145/2939672.2939754. PMC5108654 27853626

[37] Barton NH . Why species and subspecies? Curr Biol 1993;3:797–9. 10.1016/0960-9822(93)90036-N. 15335852

[38] Li W, Godzik A. CD-HIT: a fast program for clustering and comparing large sets of protein or nucleotide sequences. Bioinformatics 2006;22:1658–9. 10.1093/bioinformatics/btl158. 16731699

[39] Bin Y, Zhang W, Tang W. et al. Prediction of neuropeptides from sequence information using ensemble classifier and hybrid features. J Proteome Res 2020;19:3732–40. 10.1021/acs.jproteome.0c00276. 32786686

[40] Yu Q, Zhang Z, Liu G. et al. ToxGIN: an In silico prediction model for peptide toxicity via graph isomorphism networks integrating peptide sequence and structure information. Brief Bioinform 2024;25:bbae583. 10.1093/bib/bbae583. 39530430 PMC11555482

[41] Gawde U, Chakraborty S, Waghu FH. et al. CAMPR4: a database of natural and synthetic antimicrobial peptides. Nucleic Acids Res 2023;51:D377–83. 10.1093/nar/gkac933. 36370097 PMC9825550

[42] Tarekegn A, Giacobini M, Michalak K. A review of methods for imbalanced multi-label classification. Pattern Recogn 2021;118:107965. 10.1016/j.patcog.2021.107965.

[43] Tran TO, Le NQK. Sa-TTCA: an SVM-based approach for tumor T-cell antigen classification using features extracted from biological sequencing and natural language processing. Comput Biol Med 2024;174:108408. 10.1016/j.compbiomed.2024.108408. 38636332

[44] Le NQK . Leveraging transformers-based language models in proteome bioinformatics. Proteomics 2023;23:e2300011. 10.1002/pmic.202300011. 37381841

[45] Bhatt D, Patel C, Talsania H. et al. CNN variants for computer vision: history, architecture, application, challenges and future scope. Electronics 2021;10:2470. 10.3390/electronics10202470.

[46] Van Houdt G, Mosquera C, Nápoles G. A review on the long short-term memory model. Artif Intell Rev 2020;53:5929–55. 10.1007/s10462-020-09838-1.

[47] Beck M, Pöppel K, Spanring M. et al. xLSTM: extended long short-term memory. Adv Neural Inf Proces Syst 2024;37:107547–603.

[48] Vaswani A, Shazeer N, Parmar N. et al. Attention is all you need. NIPS 2017;NIPS'17:6000–10.

[49] Peng Z . PTM-mamba: A PTM-aware protein language model with bidirectional gated mamba blocks. ACM CIKM 2024;CIKM'24:5475–8. 10.1145/3627673.3680276. PMC12074982 40211004

[50] Schmirler R, Heinzinger M, Rost B. Fine-tuning protein language models boosts predictions across diverse tasks. Nat Commun 2024;15:7407. 10.1038/s41467-024-51844-2. 39198457 PMC11358375

[51] Martinez-Mauricio KL, Garcia-Jacas CR, Cordoves-Delgado G. Examining evolutionary scale modeling-derived different-dimensional embeddings in the antimicrobial peptide classification through a KNIME workflow. Protein Sci 2024;33:e4928. 10.1002/pro.4928. 38501511 PMC10949403

[52] Ahmed M, Seraj R, Islam SMS. The k-means algorithm: a comprehensive survey and performance evaluation. Electronics 2020;9:1295. 10.3390/electronics9081295.

[53] Nayak J, Naik B, Behera HS. Fuzzy C-means (FCM) clustering algorithm: A decade review from 2000 to 2014. ICCIDM 2014;CIDM'14:133–49. 10.1007/978-81-322-2208-8_14.

[54] Zhang Y, Li M, Wang S. et al. Gaussian mixture model clustering with incomplete data. ACM Trans Multimed Comput Commun Appl 2021;17:1–14. 10.1145/3408318.

[55] Murtagh F, Contreras P. Algorithms for hierarchical clustering: an overview. Wiley Interdisciplinary Reviews: Data Mining and Knowledge Discovery 2012;2:86–97.

[56] Zhao F, Ai Q, Li X. et al. TLC-xml: transformer with label correlation for extreme multi-label text classification. Neural Process Lett 2024;56:25. 10.1007/s11063-024-11460-z.

[57] García-Pedrajas NE, Cuevas-Muñoz JM, Cerruela-García G. et al. A thorough experimental comparison of multilabel methods for classification performance. Pattern Recogn 2024;151:110342. 10.1016/j.patcog.2024.110342.

[58] Boutell MR, Luo J, Shen X. et al. Learning multi-label scene classification. Pattern Recogn 2004;37:1757–71. 10.1016/j.patcog.2004.03.009.

[59] García-Jacas CR, Pinacho-Castellanos SA, García-González LA. et al. Do deep learning models make a difference in the identification of antimicrobial peptides? Brief Bioinform 2022;23:bbac094. 10.1093/bib/bbac094. 35380616

[60] García-Jacas CR, García-González LA, Martinez-Rios F. et al. Handcrafted versus non-handcrafted (self-supervised) features for the classification of antimicrobial peptides: complementary or redundant? Brief Bioinform 2022;23:bbac428. 10.1093/bib/bbac428. 36215083

[61] Coates A, Ng AY. *Learning feature representations with k-means*, LNTCS 2012;7700:561–80. 10.1007/978-3-642-35289-8_30.

[62] Cohen-Addad V, Esfandiari H, Mirrokni V. et al. Improved approximations for Euclidean k-means and k-median, via nested quasi-independent sets. STOC 2022;STOC'22:1621–28. 10.1145/3519935.3520011.

[63] Pang Y, Yao L, Xu J. et al. Integrating transformer and imbalanced multi-label learning to identify antimicrobial peptides and their functional activities. Bioinformatics 2022;38:5368–74. 10.1093/bioinformatics/btac711. 36326438 PMC9750108

[64] Hasannejad-Asl B, Heydari S, Azod F. et al. Peptide-membrane docking and molecular dynamic simulation of in silico detected antimicrobial peptides from Portulaca oleracea’s transcriptome. Probiotics Antimicro 2024;16:1501–15. 10.1007/s12602-024-10261-z. 38704476

[65] Chakraborty A, Kobzev E, Chan J. et al. Molecular dynamics simulation of the interaction of two linear battacin analogs with model gram-positive and gram-negative bacterial cell membranes. ACS Omega 2020;6:388–400. 10.1021/acsomega.0c04752. 33458490 PMC7807746

[66] Cao Z, Shi Z, Tong M. et al. Synergistic antimicrobial mechanism of the ultrashort antimicrobial peptide R3W4V with a tadpole-like conformation. J Chem Inf Model 2024;64:6838–49. 10.1021/acs.jcim.4c01100. 39186796

[67] Lamiable A, Thévenet P, Rey J. et al. PEP-FOLD3: faster de novo structure prediction for linear peptides in solution and in complex. Nucleic Acids Res 2016;44:W449–54. 10.1093/nar/gkw329. 27131374 PMC4987898

[68] Jo S, Kim T, Iyer VG. et al. CHARMM-GUI: a web-based graphical user interface for CHARMM. J Comput Chem 2008;29:1859–65. 10.1002/jcc.20945. 18351591

[69] Abraham MJ, Murtola T, Schulz R. et al. GROMACS: high performance molecular simulations through multi-level parallelism from laptops to supercomputers. SoftwareX 2015;1-2:19–25. 10.1016/j.softx.2015.06.001.

[70] Humphrey W, Dalke A, Schulten K. VMD: visual molecular dynamics. J Mol Graph 1996;14:33–8. 10.1016/0263-7855(96)00018-5. 8744570

