# Supplementary material for: Pepxml: ESM2-based extreme multilabel classification of pathogen-targeted antimicrobial peptides
Source: Brief Bioinform. 2025 Oct 17;26(5):bbaf548. doi: 10.1093/bib/bbaf548 (PMC12531984; doi:10.1093/bib/bbaf548)
Supplement: PepXML-Supplementary_bbaf548 [file pepxml-supplementary_bbaf548.docx]

**PepXML: ESM2-based extreme multi-label classification of pathogen-targeted antimicrobial peptides**

Yannan Bin^1,#^, Daijun Zhang^1,#^, Zhiyang Hu^2^, Chungui Xu^3,*^, Yansen Su^4,*^

^1^ The Key Laboratory of Intelligent Computing and Signal Processing of Ministry of Education, Institutes of Physical Science and Information Technology, Anhui University, Hefei, Anhui 230601, China.

^2^School of Artificial Intelligence and Automation, Huazhong University of Science and Technology, Wuhan, Hubei 430070, China.

^3^Department of Orthopaedics, the Second Affiliated Hospital of Anhui Medical University, Hefei, Anhui 230601, China.

^4^School of Artificial Intelligence, Anhui University, Hefei, Anhui 230601, China.

#These authors contributed equally to this work.

*Corresponding authors: xcg1984@bjmu.edu.cn (C. X.) and suyansen@ahu.edu.cn (Y. S.).

# Supplementary Information:

**Contents**

1. **Supplementary Methods**

**Method S1.1**: Measures of label imbalance S-3

**Method S1.2**: Deep learning algorithms S-3~4

**Method S1.3**: Clustering methods S-4~5

**Method S1.4**: Interaction Attention S-5~6

**Method S1.5**: Label Interaction Learning S-6~7

**2 Supplementary Figures**

**Figure S1**: Data processing process. S-8

**Figure S2**: Length distribution of peptide sequences in the multi-label dataset S-8

**Figure S3**: The IRLbl values of the benchmark dataset and independent test set. S-9

**Figure S4**: Architectures of large language model S-9

**Figure S5**: Visualization of label clusters with different clustering methods S-10

**Figure S6**: Performance comparison of different models S-10

**3 Supplementary Tables**

**Table S1**: Types of microbial and the number of pathogens in each type. S-11

**Table S2**: Measures of label imbalance on the benchmark dataset and independent test set. S-11

**Table S3**: Detailed information of hyperparameters for each method. S-11

**Table S4**: Comparison of the complexity and performance for PepXML with existing methods. S-12

**Table S5**: The bilayer and peptide-bilayer system composition. S-12

### Supplementary Methods

### Measures of label imbalance

1. **Imbalance ratio per label (IRLbl)**: Let *M* represent an XML dataset, which consists of a set of labels *L*. For each instance *i*, denote the label set as *Y_i_*. The imbalance Ratio for a label *λ*, denoted as IRLbl, is calculated as the ratio between the majority label and label *λ* [[1](#_ENREF_1)]. In this work, IRLbl equals 1 for the most frequent label, where other labels receive higher values. Therefore, a larger IRLbl indicates a higher level of imbalance for the specified label.

$$IRLbl\left( \lambda\right)=\frac{\max_{\lambda^{'}\in L} \left( \sum_{i=1}^{m} h\left( \lambda^{'}, Y_{i} \right) \right)}{\sum_{i=1}^{m} h\left( \lambda, Y_{i} \right)}, h\left( \lambda^{'}, Y_{i} \right)=\left\{ \begin{aligned} 1 \lambda\in Y_{i} \\ 0 \lambda\in/Y_{i} \end{aligned} \right.$$

2. **Mean imbalance ratio (MeanIR)**: It is the mean imbalance ratio among all labels in an XML dataset [[1](#_ENREF_1)].

$$MeanIR=\frac{1}{q}\sum_{\lambda^{'}\in L} IRLbl(\lambda)$$

3. **Maximum IRLbl (MaxIR)**: The ratio of the most common label against the rarest one [[1](#_ENREF_1)].

$$MaxIR= \max_{\lambda\in L} IRLbl(\lambda)$$

4. **Coefficient of variation of IRLbl (CVIR)**: CVIR measures the variation of IRLbl across all labels [[1](#_ENREF_1)]. It assesses the similarity of imbalance levels among the labels. A higher CVIR value indicates greater differences in imbalance levels among the labels, while a lower value suggests the labels experience a similar level of imbalance.

$$CVIR= \frac{IRLbl\sigma}{MeanIR}, IRLbl\sigma= \sqrt{\sum_{\lambda\in L} \frac{{(IRLbl-MeanIR)}^{2}}{q-1}}$$

### Deep learning algorithms

1. **Convolutional neural networks (CNN)**

CNN is widely recognized for their effectiveness in modeling biological sequences, leveraging local receptive fields and weight-sharing mechanisms to extract local features from these sequences [[2](#_ENREF_2)]. In comparison to traditional handcrafted features, such as amino acid composition, AAindex, and BLOSUM62 , CNN is capable of automatically and effectively identifying latent spatial features of sequences, thereby reducing the need for manual intervention and improving predictive performance. In this study, we utilized CNN to extract 128-dimensional features from peptide sequences.

1. **Long short-term memory (LSTM)**

LSTM and their variants are particularly adept at managing long-term dependencies in data [[3](#_ENREF_3)]. Specifically, BiLSTM enhance sequential information extraction by utilizing both forward and backward propagation, while mLSTM increases memory capacity to better model long sequences. sLSTM optimizes network architecture to improve adaptability to complex protein sequences, and xLSTM integrates multiple LSTM variants to enhance information capture [[4](#_ENREF_4)]. In this study, we extracted 128-dimensional feature from peptide sequences using LSTM, BiLSTM, mLSTM, sLSTM, xLSTM, respectively.

**(3) Transformer**

The Transformer model employs a self-attention mechanism that effectively captures long-range dependencies while offering robust parallel computing capabilities [[5](#_ENREF_5)]. In this study, we implements a multi-layer Transformer encoder comprising four layers, eight attention heads, and a hidden dimension set to 512, to learn the global features of AMPs.

### Clustering methods

1. **K-Means**

K-Means [[6](#_ENREF_6)] is a widely utilized iterative clustering algorithm that employs Euclidean distance as a similarity metric. The central objective of the K-Means is to minimize the sum of squared error (SSE) within each cluster. This minimization is achieved by determining the centroid of each cluster, which serves as the representative point for the cluster. The formal representation of the SSE is as follows:

where c is the number of clusters, n is the number of samples in cluster $C_{k}$, $C_{k}$ is the *k*-th cluster, $x_{i}$ represents the i-th sample in the cluster $C_{k}$, and $\mu_{k}$ is the centroid sample in the cluster $C_{k}$. Through the elbow method, the optimal number of clusters was set to 10 ($k=10$). The visualization of the label clusters based on K-Means is shown in Supplementary Figure S4(a).

1. **Fuzzy C-Means (FCM)**

FCM employs an objective function (OF) to assign data points to multiple clusters, allowing each point to belong to more than one cluster with a certain degree of fuzzy membership [[7](#_ENREF_7)]. The algorithm works by minimizing OF through an iterative process of updating the partition matric. The formal representation of the OF is articulated as follows:

where $u_{ik}$ represents the membership value of data point $x_{i}$ belonging to cluster $C_{k}$, *m* $\in(1,+\infty$) is the fuzzification parameter. The final cluster assignment is determined using the maximum membership principle, and the optimal number of clusters is set to $k=2$, and the label distribution is shown in Supplementary Figure S4(b).

1. **Gaussian mixture model (GMM)**

GMM assumes that the dataset consists of multiple Gaussian distributions, where each cluster follows a Gaussian distribution [[8](#_ENREF_8), [9](#_ENREF_9)]. The probability density function is given by:

where $\pi_{k}$ is the mixture coefficient of cluster *k*, satisfying $\sum_{k=1}^{C} \pi_{k}=1, \pi_{k}\geq0$; $\mathcal{N}\left( x | \mu_{k}, \Sigma_{k} \right)$ is the probability density function of the Gaussian distribution of cluster *k.*

where $\mu_{k}$ is the mean of the Gaussian distribution, $\Sigma_{k}$ is the covariance matrix. Ultimately, the data point can be assigned to the cluster with the highest probability. The optimal number of clusters was determined to be $C=8$, and the label distribution is illustrated in Supplementary Figure S4(c).

1. **Hierarchical clustering**

Hierarchical clustering initiates the process by treating each data point as a distinct cluster [[10](#_ENREF_10)]. It subsequently employs an individual approach to merge the most similar clusters until a predefined number of clusters are achieved. In this work, we utilized Ward’s method, which is designed to minimize the within-cluster variance during the merging process. The optimal number of clusters was determined to be $C=4$, and the label distribution corresponding to this clustering is shown in Supplementary Figure S4(d).

- 1. **Interaction attention (IA)**

IA Mechanism in XML tasks, a significant challenge lies in effectively capturing the fine-grained interaction information between input sequences and their corresponding labels. To address this issue, we propose an interaction attention mechanism that maps both peptides and target-specific pathogen labels into a shared latent semantic space. This mechanism computes interaction similarity through the dot product operation, thereby facilitating the construction of peptide representations that are sensitive to the characteristics of target-specific pathogens [[11](#_ENREF_11)]. The subsequent sections will provide a comprehensive introduction to each component of this algorithm, including its mathematical formulations.

For input peptide sequences $P\in\mathbb{R}^{n\times p}$ and label set $L\in\mathbb{R}^{k\times l}$ (where, *n* represents the number of peptides, *p* represents the embedding dimension of the peptide, *k* represents the number of labels, and *l* represents the embedding dimension of the labels), we first map them into an *r*-dimensional latent space via an embedding function to obtain the corresponding embedding:

where $P_{emb}\in\mathbb{R}^{n\times r}$ represents peptides mapped to potential space size, and $L_{emb}\in\mathbb{R}^{k\times r}$ represents labels mapped to potential space size.

To align the label embedding with the peptide embedding in the same space, a bridging transformation matrix $W_{bridge}\in\mathbb{R}^{r\times r}$ is designed to perform a linear transformation on the label embedding, yielding the query representation of the labels in the peptide embedding space:

where $Q\in\mathbb{R}^{r\times k}$ is the label query matrix.

Based on the interaction between the peptide embedding and the label query representations, an interaction score matrix $S\in\mathbb{R}^{n\times k}$ is computed using the dot product. Let the embedding of the *i*-th peptide be $P_{emb}^{(i)}\in\mathbb{R}^{r}$, and the query vector corresponding to the *j*-th label be $Q^{(j)}\in\mathbb{R}^{r}$; then, the interaction score is defined as:

Overall, this can be expressed as a matrix multiplication:

To obtain a reasonable attention distribution, the *softmax* function is applied to the interaction scores $S_{ij}$ for each label *j*, yielding the attention weights $A\in\mathbb{R}^{n\times k}$:

Finally, by performing a weighted sum of the peptide embedding according to the attention weights, the label-aware peptide representation is obtained. Specifically, for the *j*-th label, its corresponding representation $C^{(j)}$ is defined as:

Or expressed in matrix form as:

where $C\in\mathbb{R}^{r\times k}$ is the final label-aware peptide representation.

**1.5 Label Interaction Learning (LIL)**

In extreme multi-label classification tasks, labels often have inherent correlations. Traditional classification methods, such as simple fully connected layers, tend to overlook these inter-label relationships, thereby failing to fully capture the interaction information between labels. To effectively leverage the correlations between labels, we propose the LIL method [[12](#_ENREF_12)]. LIL introduces a label interaction module to learn the importance of different labels and adaptively adjusts the prediction of labels based on their dependencies.

LIL predicts the raw output LIL(0) through a fully connected layer and then aggregates the original output with the information from adjacent labels using a residual mapping. The specific process is as follows:

where $\Phi(x_{i})$ is the feature representation of the input sample $x_{i}$, $W_{c}$ is the learning parameter matrix that adjusts the neighboring label information, $A^{r}$ is the conditional probability matrix between labels, representing the co-occurrence relationships between labels.

The LIL algorithm is trained using the cross-entropy loss function to optimize the label prediction results:

where $y_{il}$ represents the true label and $\hat{y}_{il}$ represents the predicted label. This loss function is used to train the LIL algorithm.

**References**

1. Tarekegn, A., M. Giacobini, and K. Michalak, *A review of methods for imbalanced multi-label classification.* Pattern Recogn., 2021. **118**: 107965.

2. Bhatt, D., et al., *CNN variants for computer vision: history, architecture, application, challenges and future scope.* Electronics, 2021. **10**(20): 2470.

3. Mienye, I.D., T.G. Swart, and G. Obaido, *Recurrent neural networks: A comprehensive review of architectures, variants, and applications.* Information, 2024. **15**(9): 517.

4. Beck, M., et al., *xLSTM: Extended long short-term memory.* arXiv preprint arXiv:2405.04517, 2024.

5. Vaswani, A., et al. *Attention is all you need*. in *31st Annual Conference on Neural Information Processing Systems (NIPS)*. 2017. Long Beach, CA.

6. Ahmed, M., R. Seraj, and S.M.S. Islam, *The k-means algorithm: a comprehensive survey and performance evaluation.* Electronics, 2020. **9**(8): 1295.

7. Nayak, J., B. Naik, and H.S. Behera. *Fuzzy C-Means (FCM) clustering algorithm: a decade review from 2000 to 2014*. in *1st International Conference on Computational Intelligence in Data Mining (ICCIDM)*. 2015. Veer Surendra Sai Univ Technol, Dept Comp Sci & Engn, Informat Technol, Burla, INDIA.

8. Zhang, Y., et al., *Gaussian mixture model clustering with incomplete data.* ACM Transactions on Multimedia Computing Communications and Applications, 2021. **17**(1): 1-14.

9. Reynolds, D.A., *Gaussian mixture models.* Encyclopedia of Biometrics, 2009. **741**(659-663): 3.

10. Murtagh, F. and P. Contreras, *Algorithms for hierarchical clustering: an overview.* Wiley Interdisciplinary Reviews: Data Mining and Knowledge Discovery, 2012. **2**(1): 86-97.

11. Huang, X., et al., *Label-aware document representation via hybrid attention for extreme multi-label text classification.* Neural Process. Lett., 2022: 1-17.

12. Zhao, F., et al., *TLC-XML: transformer with label correlation for extreme multi-label text classification.* Neural Process. Lett., 2024. **56**(1): 25.

**2 Supplementary Figures**


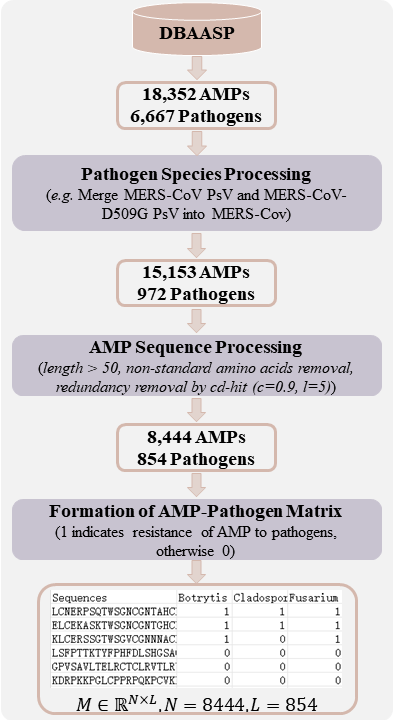


**Figure S1:** Data processing process.


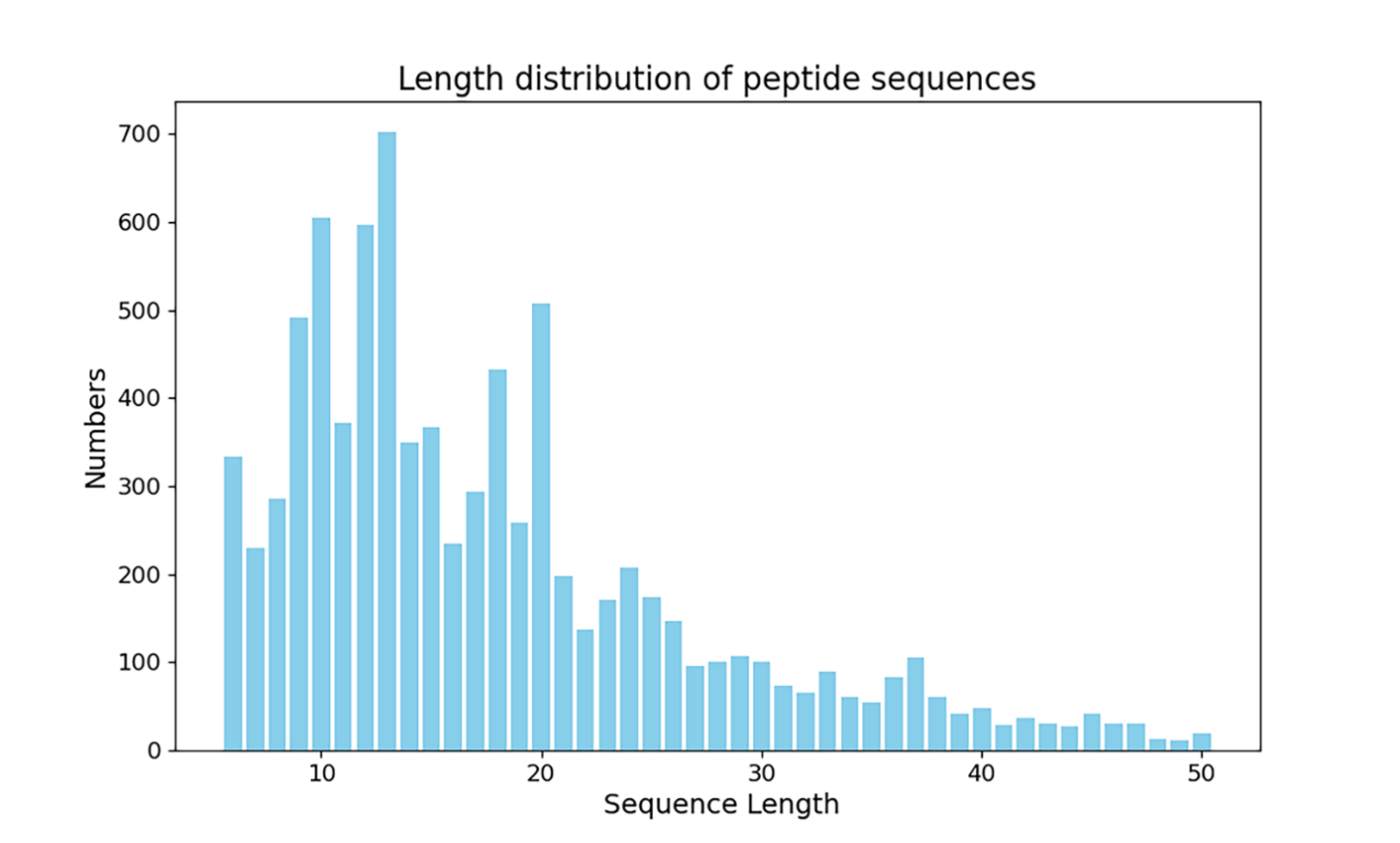


**Figure S2:** Length distribution of peptide sequences in the multi-label dataset.


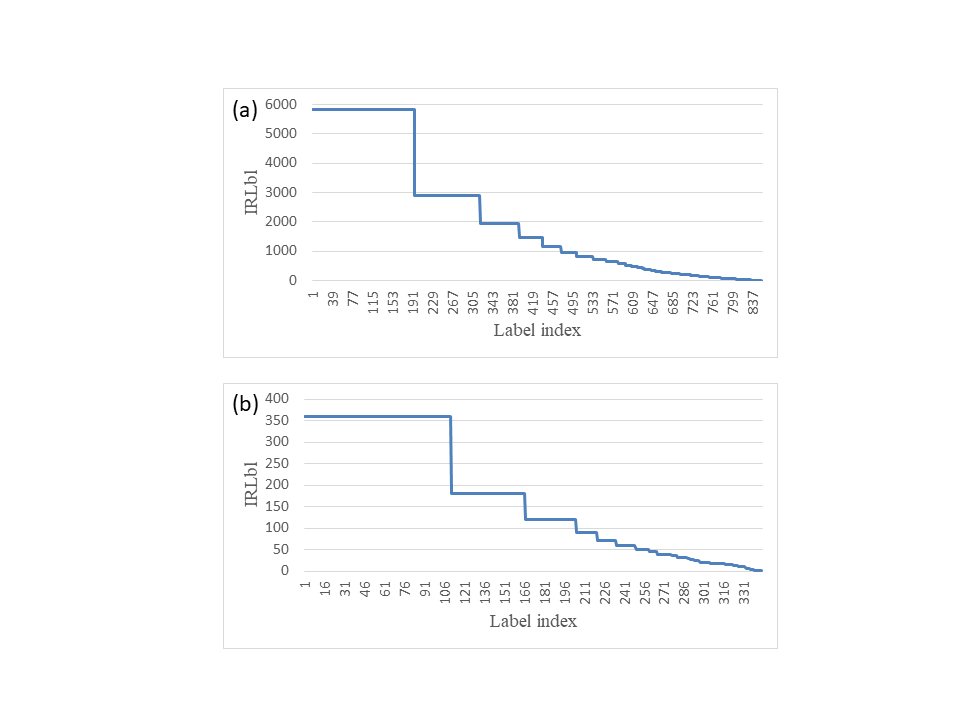


**Figure S3** The IRLbl values of the benchmark dataset (a) and independent test set (b).


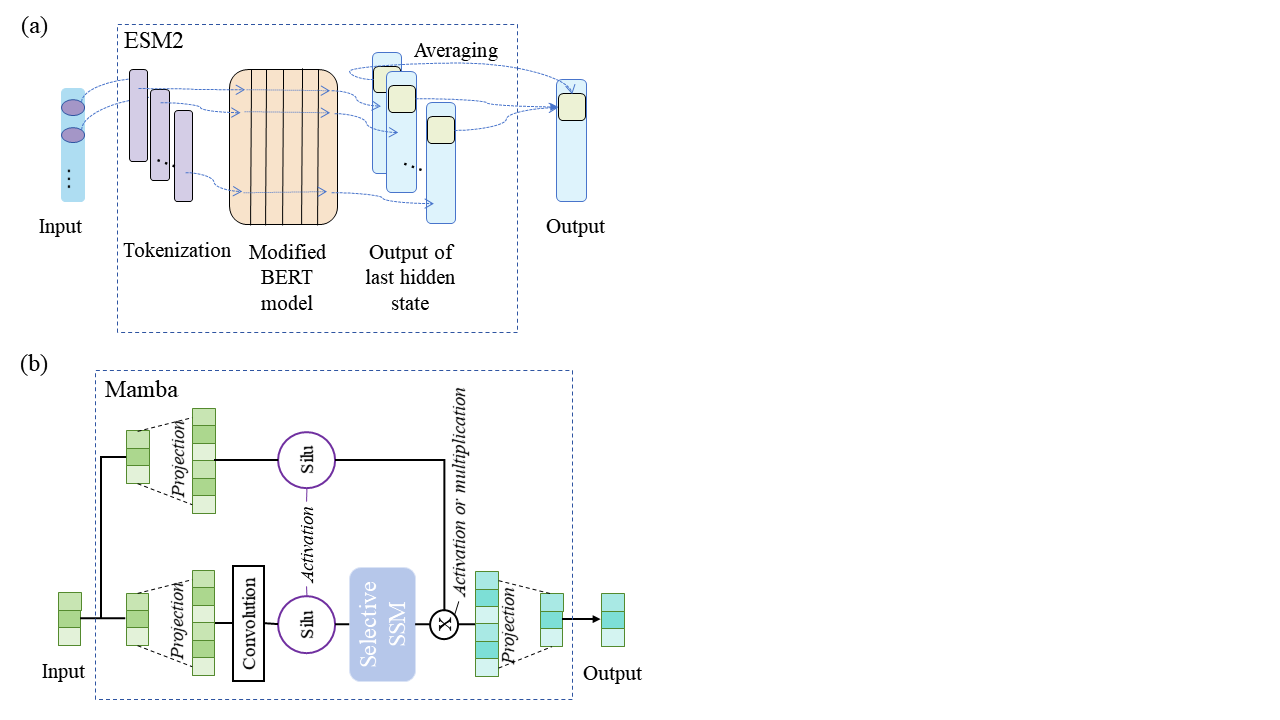


**Figure S4:** Architectures of large language model. (a) ESM2 and (b) Mamba.


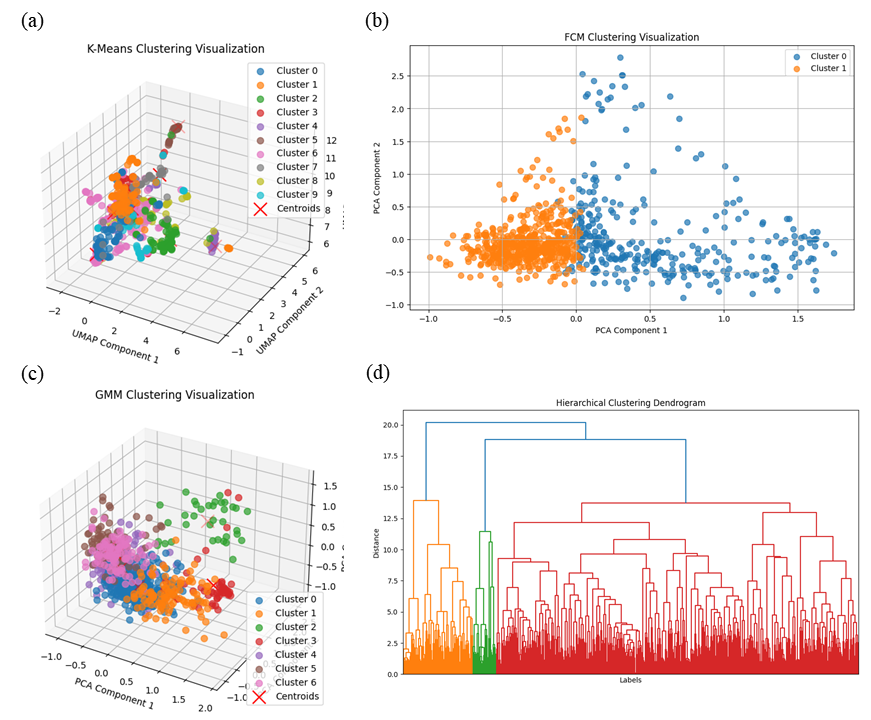


**Figure S5:** Visualization of label clusters with different clustering methods.


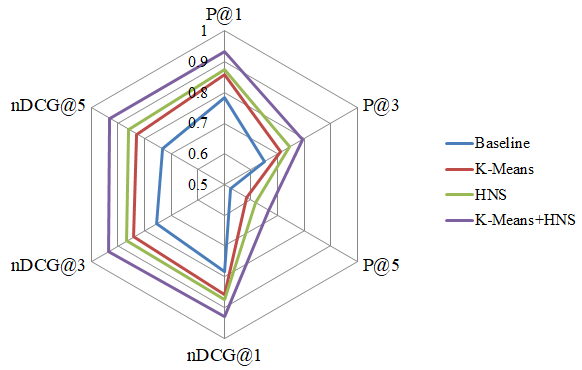


**Figure S6:** Performance comparison of different models (baseline, K-Means, HNS and K-Means+HNS).

**3 Supplementary Tables**

**Table S1**: Types of microbial and the number of pathogens in each type.

| NO. | Type | Number of pathogens |
| --- | --- | --- |
| 1 | Bacteria | 458 |
| 2 | Fungus | 207 |
| 3 | Cancer | 82 |
| 4 | Virus | 59 |
| 5 | Parasite | 28 |
| 6 | Mycoplasma | 9 |
| 7 | Oomycota | 11 |

**Table S2**: Measures of label imbalance on the benchmark dataset and independent test set.

| Measure | Benchmark dataset | Independent test set |
| --- | --- | --- |
| MeanIR | 2208 | 175 |
| MaxIR | 5813 | 361 |
| CVIR | 0.974 | 0.786 |

**Table S3:** Detailed information of hyperparameters for each method.

| Methods | Learning Rate | Batch Size | Epochs | Embedding Dimension | Dropout |
| --- | --- | --- | --- | --- | --- |
| PepXML | 0.001 | 32 | 100 | 1280 | 0.3 |
| PrMFTP | 0.001 | 64 | 100 | 128 | 0.6 |
| TransImbAMP | 0.04 | 32 | 256 | 768 | 0.2 |
| ETFC | 0.0018 | 192 | 200 | 192 | 0.6 |

**Table S4:** Comparison of the complexity and performance for PepXML with existing methods.

| Model | FLOPs↓ | Parameters↓ |
| --- | --- | --- |
| ETFC | 6.209×10^9^ | 2.439×10^7^ |
| TransImbAMP | 1.463×10^10^ | 9.279×10^7^ |
| PrMFTP | 1.920×10^7^ | 1.239×10^6^ |
| ESM2 (baseline model) | 1.102×10^10^ | 1.510×10^8^ |
| PepXML (our methods) | 1.103×10^10^ | 6.516×10^8^ |

**Table S5**: The bilayer and peptide-bilayer system composition.

| Membrane | Lipids | | | | Water | SOD | CLA | Ion concentration | Lipid area | Peptide area | Distance |
| --- | --- | --- | --- | --- | --- | --- | --- | --- | --- | --- | --- |
|  | POPE | POPG | TOCL1 | POPC |  |  |  |  |  |  |  |
| *E. coli* | 62 | 12 | 4 | 0 | 2.25 | 23 | 11 | 0.15 | 2447 | 36.6 | 3nm |
| *S. aureus* | 0 | 46 | 32 | 0 | 2.25 | 90 | 16 | 0.15 | 3443.6 | 36.6 | 3nm |

Note: POPE, palmitoyloleoyl phosphatidylethanolamine. POPG, palmitoyloleoyl phosphatidylglycerol. TOCL1, lipids cardiolipin. POPC, palmitoyloleoyl phosphatidylcholine. SOD, superoxide dismutase. CLA, conjugated linoleic acid.
